# When Rewiring Fails—The Enduring Role of the Pectoralis Major Flap in Sternal Wound Reconstruction

**DOI:** 10.3390/jcm14238376

**Published:** 2025-11-26

**Authors:** Viktoria Koenig, Alexandra Christ, Maximilian Monai, Martin Andreas, Daniel Zimpfer, Wolfgang Happak, Paul Werner

**Affiliations:** 1 Division of Plastic, Aesthetic and Reconstructive Surgery, Medical University of Vienna, Waehringer Guertel 18-20, 1090 Vienna, Austria; alexandra.christ@meduniwien.ac.at (A.C.); n11927646@students.meduniwien.ac.at (M.M.); wolfgang.happak2@wienerneustadt.lknoe.at (W.H.); 2 Division of Cardiac Surgery, Medical University Graz, Neue Stiftingtalstraße 6, 8010 Graz, Austria; dr.andreas@me.com; 3 Division of Cardiac Surgery, Medical University of Vienna, Waehringer Guertel 18-20, 1090 Vienna, Austria; daniel.zimpfer@meduniwien.ac.az (D.Z.); paul.werner@meduniwien.ac.at (P.W.)

**Keywords:** deep sternal wound infection (DSWI), cardiac surgery, pectoralis major flap, sternal dehiscence

## Abstract

**Background**: Deep sternal wound infections (DSWIs) remain a serious complication after median sternotomy, often requiring complex wound management strategies. While modern approaches include vacuum-assisted closure (VAC) and plating techniques, the pedicled pectoralis major muscle flap (PMF) continues to play a pivotal role in surgical reconstruction, especially in cases with sternal destruction or osteomyelitis. **Methods**: In this retrospective single-centre analysis, 166 patients with DSWI following cardiac surgery were reviewed. Clinical data, comorbidities, laboratory parameters, and surgical management were evaluated. Logistic regression was performed to assess predictors for reinfection and need for reoperation. **Results**: Initial wound revision was most frequently performed using sternal rewiring (60.2%), followed by reconstruction with a pectoralis major flap (33.7%). Despite initial surgical treatment, 27.1% of patients developed post-revision wound healing disturbances, and 24.1% ultimately required a second surgical intervention. Among second-time procedures, VAC therapy (32.5%) and PMF reconstruction (20.0%) were the most common approaches. Reinfection was significantly associated with higher preoperative EuroSCOREs (*p* = 0.044), while initial rewiring carried a higher risk of treatment failure compared to the pectoralis major flap (*p* = 0.0024). **Conclusions**: In the setting of sternal destruction or osteomyelitis, the pectoralis major muscle flap remains a fast, effective, and robust solution. Despite its long-standing use, it continues to offer excellent vascularized coverage and infection control in complex DSWI cases.

## 1. Introduction

Deep sternal wound infection (DSWI) is a rare but serious complication after cardiac surgery via median sternotomy, with an incidence of 0.2–6% [1,2,3,4]. Despite its low frequency, DSWI is linked to high morbidity, prolonged hospitalization, reoperation rates, and mortality exceeding 25% in severe cases [4,5,6]. Effective management requires timely diagnosis, interdisciplinary collaboration, and individualized treatment strategies [7,8,9,10].

The multifactorial pathogenesis involves sternal instability, impaired perfusion, and bacterial contamination [10,11,12,13]. Common pathogens include Staphylococcus aureus, Staphylococcus epidermidis, and Gram-negative organisms, with rising multidrug resistance [14]. Risk factors such as diabetes, obesity, COPD, renal dysfunction, immunosuppression, prolonged surgery, bilateral mammary artery use, and re-exploration increase susceptibility [15,16,17].

Clinically, DSWI presents with sternal dehiscence, purulent drainage, or systemic signs, and may progress to osteomyelitis or mediastinitis [18]. Diagnosis relies on clinical, microbiological, and imaging findings [14,19]. Treatment includes radical debridement, targeted antibiotics, and coverage with well-vascularized flaps [20,21,22,23,24,25,26,27,28,29,30,31]. Negative pressure wound therapy (VAC) serves as an important adjunct for wound conditioning and reconstruction bridging [32]. We identified impaired antibiotic penetration from mammary artery harvesting and showed that early prophylaxis and direct sternal antibiotic application improve tissue levels and reduce infection risk [33,34].

Deep sternal wound infection (DSWI) remains a severe complication after cardiac surgery, associated with prolonged hospitalization and high mortality. While rewiring or vacuum-assisted closure may suffice in selected cases, many patients present with sternal destruction or underlying osteomyelitis, requiring vascularized soft tissue coverage for definitive healing. The pedicled pectoralis major flap (PMF) offers reliable coverage and infection control in such complex cases, yet data on long-term outcomes and clinical predictors for flap reconstruction remain limited.

This study analyzes 166 cases of sternal dehiscence or DSWI after cardiac surgery. The aim was to assess outcomes, identify predictors of reinfection and reoperation, and highlight the role of the PMF as a durable reconstructive strategy in patients with advanced sternal involvement.

## 2. Materials and Methods

### 2.1. Study Design

This retrospective, single-centre cohort study was conducted at a tertiary academic level-one referral centre providing cardiac and plastic–reconstructive surgery services. Ethical approval was obtained from the institutional review board (No. 1660/2020).

### 2.2. Setting and Population

The hospital serves a large metropolitan and national referral population and performs approximately 800–900 open-heart procedures in adult patients annually. The cardiac surgery and reconstructive surgery units closely cooperate in managing complex postoperative wound complications, including deep sternal wound infection (DSWI).

### 2.3. Eligibility Criteria

Eligible were all patients with sternal dehiscence and DSWI after full median sternotomy for cardiac surgery between 2000 and 2024. Only patients who ultimately underwent rewiring or pedicled pectoralis major flap (PMF) reconstruction as the definitive procedure for sternal wound closure were included. Wound management

Negative-pressure wound therapy (NPWT) was performed using commercially available systems (Lohmann & Rauscher or KCI). The target pressure was generally set to −250 mmHg in continuous mode, with lower settings applied in selected cases with deep sternal cavities or fragile tissue. VAC dressings were typically changed every 5–7 days, depending on wound appearance and clinical course.

Initial surgical debridement was carried out by the cardiac surgery team as part of the index revision procedure. The technique and extent of debridement were adapted to intraoperative findings and tissue condition.

Definition of DSWI: DSWI was defined according to the Centers for Disease Control and Prevention (CDC) criteria, requiring deep tissue involvement confirmed clinically, microbiologically, or radiologically.

Definition of superficial infection: Superficial infection was defined as involvement limited to skin or subcutaneous tissue without sternal instability, osteomyelitis, or mediastinal extension. Such cases were excluded.

Further exclusion criteria were alternative surgical approaches (e.g., hemisternotomy), incomplete records, and missing follow-up.

### 2.4. Variables and Outcomes

Data were extracted from operative reports, medical charts, and laboratory databases using standardized templates. Collected parameters included demographics, comorbidities, operative details, laboratory values, pathogens, VAC duration, reconstruction type (unilateral/bilateral PMF), RE-VAC, and associated procedures. Primary outcomes were in-hospital and 30-day mortality, reinfection, and wound stability. Preoperative risk assessment was based on the European System for Cardiac Operative Risk Evaluation (EuroSCORE, EuroSCORE II) and the Society of Thoracic Surgeons (STS) predicted risk scores for mortality and combined morbidity/mortality. Scores were calculated according to standard institutional practice using the parameters documented in the preoperative anesthesiology and surgical risk assessment forms. The results were included as continuous variables in the statistical analysis.

Definition of reinfection: Reinfection was defined as recurrence of deep wound infection fulfilling CDC criteria within 90 days after closure, requiring antibiotic therapy and/or surgical revision.

Definition of wound stability: Wound stability was defined as absence of sternal motion, dehiscence, or drainage at 30-day follow-up, assessed clinically by the treating surgeon.

No structured wound assessment score (e.g., RESVECH, RYB) was used, as such tools were not consistently available throughout the study period. This limitation is discussed accordingly.

Patients were categorized according to their definitive closure technique. Those who initially underwent rewiring but later required pectoralis major flap (PMF) reconstruction were analyzed within the PMF group. Thus, group allocation reflects the final reconstructive approach rather than initial management.

### 2.5. Statistical Analysis

Group comparisons were performed for rewiring versus PMF and for reinfection versus no reinfection. Continuous variables were analyzed using Student’s *t*-test or Mann–Whitney U test, depending on data distribution. Categorical variables were compared with the Chi-square or Fisher’s exact test. Effect sizes (mean difference with 95% CI or odds ratios) were calculated where appropriate. Normality was assessed using the Shapiro–Wilk test. Categorical variables were compared using Chi-square or Fisher’s exact tests, and continuous variables were compared with Student’s *t*-test or Mann–Whitney U test, as appropriate. Data are reported as mean ± standard deviation or median [range]. A *p*-value < 0.05 was considered statistically significant. Multivariable logistic regression was performed to identify independent predictors of reinfection and reintervention. Variables with *p* < 0.1 in univariate testing or clinical relevance were included in the model (sex, BMI, hypertension, diabetes, renal insufficiency, smoking, EuroSCORE, and surgical method). Collinearity was assessed using variance inflation factors (VIF < 2 for all included predictors). Missing data were <5% and were handled by listwise deletion. Results are presented as adjusted odds ratios (ORs) with 95% confidence intervals (CIs). Analyses were performed using SPSS version 30.0.0.0 (IBM Corp, Armonk, New York, NY, USA) and R version 3.1.1.

## 3. Results

The study cohort included 166 patients with a mean age of 64.8 ± 11.8 years and an average body mass index (BMI) of 29.9 ± 6.1 kg/m^2^. The majority of patients were male (n = 114; 68.7%), while 52 patients (31.3%) were female. Nearly half of the cohort (48.8%) had a history of smoking. Arterial hypertension was the most prevalent comorbidity (57.8%), followed by dyslipidemia, diabetes mellitus, and coronary artery disease. Further baseline characteristics, including cardiac, pulmonary, neurological, and renal comorbidities, are summarized in Table 1.

Preoperative laboratory values reflected characteristic alterations in patients undergoing major cardiac surgery complicated by deep sternal wound infection (DSWI). Hemoglobin levels were mildly reduced (mean 12.5 ± 1.9 g/dL), particularly among female patients. Inflammatory markers were markedly elevated, with a mean C-reactive protein (CRP) of 18.3 ± 36.2 mg/dL and fibrinogen levels of 446.6 ± 144.1 mg/dL, indicating a pronounced systemic inflammatory response. Mean serum albumin was 39.7 ± 5.4 g/L. Renal function varied considerably (mean eGFR 84.3 ± 38.0 mL/min/1.73 m^2^), ranging from severe impairment to supranormal values. Coagulation parameters, liver enzymes, and electrolytes were largely within normal limits, with the exception of elevated gamma-glutamyl transferase (γ-GT) levels in male patients.

Preoperative risk assessments indicated moderate predicted morbidity and mortality, with an additive EuroSCORE of 8.3 ± 3.7 (8.2 in males, 8.5 in females), a logistic EuroSCORE of 11.9 ± 8.6%, EuroSCORE II of 5.6 ± 5.4%, STS-predicted mortality of 2.7 ± 2.8%, and STS morbidity and mortality of 12.1 ± 8.2%. A full overview of laboratory values and risk scores is provided in Table 2.

Coronary artery bypass grafting (CABG) was the most common procedure, performed in 65.1% of patients, followed by aortic surgery (34.9%) and valve interventions (19.9%). Concomitant procedures were documented in 25.3%, and 17.5% had a history of previous cardiac surgery. Within 30 days postoperatively, extracorporeal membrane oxygenation (ECMO) was required in 10.8% of cases.

The mean intensive care unit (ICU) stay was 22.3 ± 37.7 days, and the total hospital stay averaged 67.5 ± 55.4 days. Cardiopulmonary bypass (CPB) time averaged 147.7 ± 68.6 min (median 133.5), and aortic cross-clamp (ACC) time was 83.9 ± 45.2 min (median 76.0). These figures reflect the procedural complexity and high morbidity associated with DSWI (Table 3).

**Figure 1:**
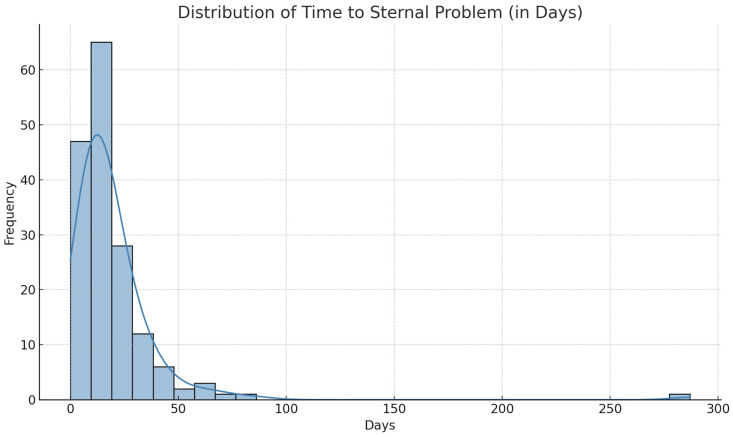
Distribution of Time to Sternal Problem. A histogram (blue bars) illustrates the number of patients developing a sternal problem at different postoperative time intervals. The overlaid blue curve represents the kernel density estimate (KDE), showing the smoothed distribution of onset times. Most sternal complications occur early after surgery, with only a few cases presenting at later time points.

The median time to sternal complication was 14 days (mean 19.2 ± 25.1; IQR 9–22), indicating that most revisions occurred within the first three postoperative weeks. However, 6.6% (11 patients) experienced very delayed complications after day 45. This right-skewed distribution is reflected by the high standard deviation and rare outliers, including one case almost 10 months post-surgery (Figure 1).

Negative pressure wound therapy (VAC) was used in 147 of 166 patients (88.6%) with DSWI or sternal dehiscence; only 19 patients (11.4%) were treated without VAC. Intravenous antibiotics were given in 155 cases (93.4%), while 11 patients (6.6%) received no i.v. therapy. Blood cultures were positive in 58 patients (34.9%), underlining the systemic infectious burden of DSWI. Pathogens were isolated in 121 wound cultures (72.9%), predominantly coagulase-negative staphylococci (~50%), including Staphylococcus epidermidis (~27%), followed by Staphylococcus aureus (~20%) and Gram-negative bacteria (~10%). The rise in multidrug-resistant strains underscores the need for targeted antibiotics.

**Figure 2:**
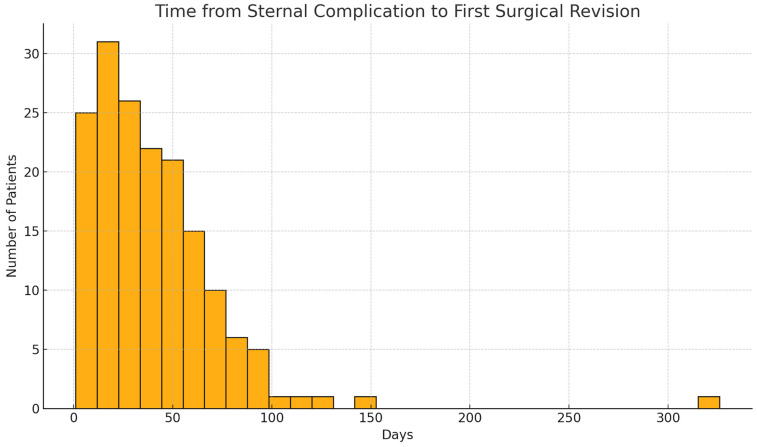
Time from Sternal Complication to First Surgical Revision.

Sternal instability was present in 86 of 166 patients (51.8%), while 80 patients (48.2%) had DSWIs without evident mechanical disruption of the sternum. The mean interval between symptom onset and first surgical revision was 40.7 ± 34.6 days (median 34.5; IQR 16.5–55.0; range 1–326). Revision surgery occurred within two weeks in 33 patients (19.9%), while 31 (18.7%) had a delay > 60 days and 9 (5.4%) > 90 days after symptom onset (Figure 2).

Initial surgical strategies after DSWI diagnosis were stratified by the first intervention performed. Rewiring was the most common definitive procedure (100 patients, 60.2%), typically used for sternal stabilization in the absence of major soft tissue defects. Pectoralis major flap reconstruction represented the second most frequent definitive approach (56 patients, 33.7%), providing vascularized coverage in cases with tissue loss, infection, or instability. In several patients, VAC therapy or debridement with cerclage removal had been applied as interim management prior to definitive closure. Debridement with cerclage removal alone was performed in 4 patients (2.4%), likely in superficial infections or where wire removal sufficed for infection control. A multivariate logistic regression identified predictors of reinfection after DSWI surgery. Independent variables included sex, BMI, hypertension, diabetes, renal insufficiency, smoking, EuroSCORE, and surgical method. The EuroSCORE emerged as a significant predictor: higher scores were associated with increased reinfection risk (*p* = 0.044), highlighting the impact of overall perioperative risk on outcomes after sternal reconstruction. Other variables such as sex, BMI, hypertension, diabetes, smoking status, and renal insufficiency did not show a statistically significant association with the risk of reinfection in this multivariate model (all *p* > 0.05).

**Figure 3:**
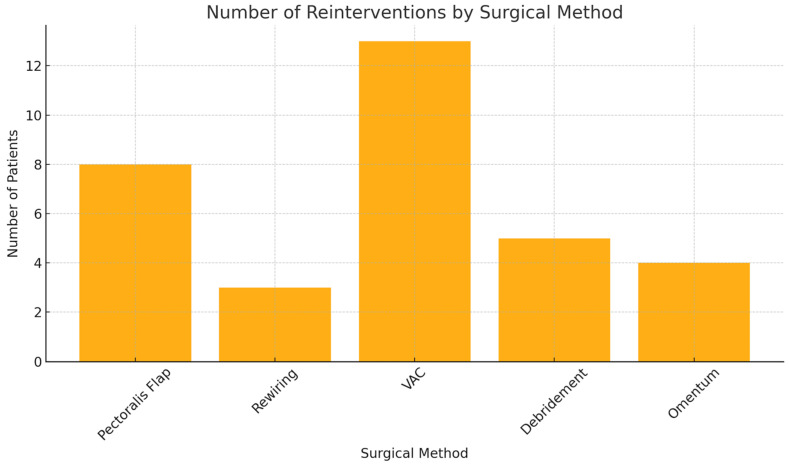
Number of Reinterventions by Surgical Method.

A total of 45 patients (27.1%) experienced reinfection or recurrent wound healing disorders after initial DSWI surgery; 40 (24.1%) required a second surgical intervention. The reconstructive approaches varied: VAC therapy was most common (13 cases, 32.5%), followed by pectoralis major muscle flaps (8 patients, 20.0%), including second flap procedures. Omental transposition was used in 4 cases (10.0%), rewiring in 3 (7.5%), and isolated debridement with cerclage removal in 5 (12.5%) (Figure 3).

Flap-based techniques (pectoralis flaps) were significantly more common in complex wound cases (*p* = 0.0001). A multivariate logistic regression analysis examined sex, BMI, hypertension, EuroSCORE, and initial surgical method as predictors of reintervention. Sex, BMI, and hypertension showed no significant associations. The EuroSCORE showed a non-significant trend (*p* = 0.223). In contrast, the surgical method used in the first revision was a strong predictor (*p* = 0.0024): patients who initially received flap coverage were significantly less likely to require further surgery than those treated with rewiring or VAC alone.

**Figure 4:**
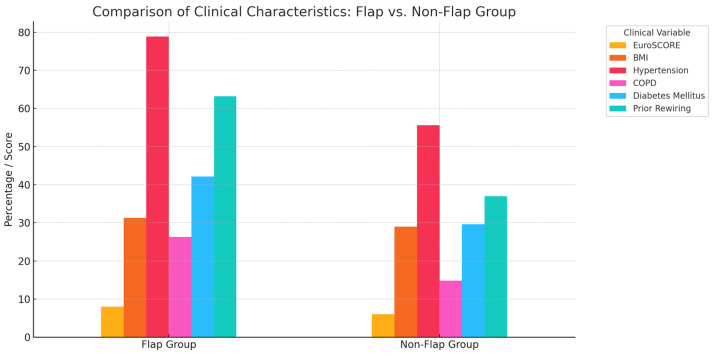
Comparison of Clinical Characteristics.

Among patients undergoing flap reconstruction in the second revision, the mean EuroSCORE was significantly higher (8.0 ± 2.6 vs. 6.0 ± 2.7; *p* = 0.021), indicating a higher preoperative risk. They also had a higher mean BMI (31.3 ± 5.2 kg/m^2^ vs. 29.0 ± 6.4 kg/m^2^; *p* = 0.181, n.s.) and more comorbidities: hypertension (78.9% vs. 55.6%, *p* = 0.074), COPD (26.3% vs. 14.8%), and diabetes (42.1% vs. 29.6%), though these differences were not statistically significant. Notably, 63.2% of flap patients had undergone rewiring in the first revision (vs. 37.0%, *p* = 0.041), suggesting treatment failure or more complex pathology requiring escalation. These data indicate that patients needing flap-based revision were sicker, with higher risk scores and more comorbidities. The need for flap coverage likely reflects both wound severity and the limitations of simpler techniques in high-risk patients (Figure 4). In the multivariable logistic regression, the initial surgical method remained the only independent predictor of reintervention (adjusted OR = 2.95; 95% CI 1.45–6.02; *p* = 0.0024). Higher EuroSCORE values were independently associated with the reinfection risk (adjusted OR = 1.21 per point; 95% CI 1.01–1.46; *p* = 0.044) (Table 4).

## 4. Discussion

This study highlights that patients with DSWI represent a severely ill cohort with high morbidity and prolonged hospital stays. Our analysis identified the initial surgical strategy as a key predictor of reintervention. Patients treated with a pectoralis major flap had significantly fewer revisions than those managed with rewiring or VAC, suggesting advantages of early radical debridement and vascularized coverage—particularly in cases of occult osteomyelitis. Traditional risk factors showed no significant association, emphasizing that procedural choice outweighed baseline patient characteristics [12,16].

In line with Liu et al. and Tang et al. our cohort (mean age 65, BMI 30 kg/m^2^) confirms that DSWI predominantly affects elderly, multimorbid patients [12,26]. Hypertension (58%) and diabetes (19%) were common, supporting Tang’s findings on vascular risk factors [12]. Zhao et al. linked HbA1c > 5.7% to a twofold increased DSWI risk post-CABG [11]. Our data confirm diabetes as a relevant contributor. Elevated CRP and hypoalbuminemia suggest a systemic inflammatory and metabolic instability, while BMI correlated with DSWI incidence, consistent with Ma et al. [10,16]. In our reinfection subgroup, the BMI distribution closely paralleled that of the overall cohort.

Renal impairment (14%) and dialysis (2%) were associated with elevated risk, in line with Yasuura et al., who reported fourfold flap-related mortality in dialysis patients, and Biancari et al., who incorporated renal dysfunction and BIMA use in the E-CABG score [17,34]. In our cohort, the preoperative EuroSCORE emerged as the only independent predictor of reinfection, reinforcing its value in perioperative risk stratification.

The median time to wound breakdown was 14 days, though 6% presented after postoperative day 45. Ma et al. noted that deep infections tend to manifest later than superficial ones, complicating early detection [16]. Our findings highlight the need for extended clinical vigilance in high-risk individuals. Early CT imaging, as recommended by Singh et al., proved essential for diagnosing sternal instability and bone involvement, which was intraoperatively confirmed in more than half of our patients [35].

Negative-pressure wound therapy (NPWT) was applied in 90% of cases, mainly as a bridge to reconstruction. Sjogren et al. reported reduced mortality with NPWT in DSWI, yet Lo Torto et al. observed increased flap failure with prolonged NPWT [36,37]. Our results support NPWT as a temporary adjunct—especially in the presence of osteomyelitis or instability, where early reconstruction should not be delayed.

Rewiring was the most common initial approach (60%), but also the strongest predictor of failure (*p* = 0.0024), mirroring Yang et al.’s findings on infection risk following repeated sternotomy [10]. Simple stabilization techniques are insufficient in cases with osteomyelitis [31,38]. Complete debridement to bleeding bone, as advocated by Khashkhusha et al., remains essential [4].

Definitive reconstruction requires vascularized soft tissue. Despite growing interest in fasciocutaneous flaps, the pedicled pectoralis major flap (PMF) remains widely used [6,20,22]. Zhang et al. emphasized its reliable anatomy and short operative time, making it particularly suitable in unstable patients [22]. In our cohort, PMF was applied in 34% of primary and 20% of salvage cases. Multivariate analysis confirmed that PMF halved reoperation risk (*p* < 0.01), consistent with Perezgrovas-Olaria et al. [6]. Bilateral flaps were reserved for advanced osteomyelitis; unilateral PMF was sufficient in most cases, supporting Lo Torto et al.’s recommendation [36].

Alternative or additive flaps were used in 10%, including omental and composite flaps. Yu et al. demonstrated favorable outcomes combining PMF with rectus-based elements [21]. The IMAP flap described by Koulaxouzidis et al. offers a less invasive option for stable cases [29]. In malnourished or dialysis-dependent patients, PMF remains preferable due to its volume and perfusion [20]. Van Wingerden et al. found no clear flap superiority, but Yasuura et al. reported higher mortality with omentoplasty in ventilated or dialysis-dependent patients [20,34].

Importantly, our data confirm that the initial surgical treatment significantly influenced reintervention rates. Common risk factors such as BMI, sex, and hypertension were not predictive, while PMF coverage significantly reduced recurrence. Early osteomyelitis may be clinically silent yet decisive, highlighting the value of timely radical debridement and vascularized reconstruction. While general risk tools like EuroSCORE offer perioperative insight, DSWI-specific models such as the E-CABG score may better guide early flap strategies.

From a health-economic perspective, DSWI imposes a substantial burden. Sears et al. showed that it nearly triples overall hospital costs and quadruples length of stay [39]. Our patients averaged 68 hospitalization days, often requiring multiple procedures, prolonged ICU stays, and long-term antibiotics. Schiraldi et al. found that early flap coverage shortened ICU and total stay [8]. Our findings support early, definitive reconstruction—particularly using PMF—as both clinically effective and cost-reducing.

In summary, DSWI demands individualized, interdisciplinary management. Recerclage or plating alone is inadequate in osteomyelitis [31]. Radical debridement, partial sternectomy, and early flap coverage remain the cornerstone of therapy [4,13]. NPWT is a valuable temporary measure but must not delay reconstruction [4,32]. In multimorbid and unstable patients, the PMF remains a robust and reliable option for durable sternal reconstruction.

## 5. Conclusions

This study examines DSWI in a high-risk, multimorbid cohort after cardiac surgery. Most infections occurred within 3 weeks, though late cases highlight the need for ongoing vigilance. VAC and antibiotics were standard; pathogens were frequently identified, with systemic signs in over one-third. Rewiring failed more often in cases with instability or osteomyelitis, while early pectoralis major flap coverage proved more durable. Flap patients had higher EuroSCOREs and comorbidities, indicating greater wound severity. One-quarter required reoperation, mostly after failed rewiring. Multivariate analysis confirmed the initial surgical strategy as a key predictor of revision, supporting early, risk-adapted intervention.

### Limitations

This study has several limitations. Its retrospective, single-centre design may introduce selection and information bias. Although the data collection was performed systematically, incomplete documentation across the extended study period led to occasional missing values. Furthermore, while multivariate logistic regression was conducted to identify independent predictors of reinfection and reintervention, the number of outcome events limited the inclusion of additional covariates. Therefore, residual confounding cannot be fully excluded. Prospective multicentre studies with standardized data collection and larger sample sizes are warranted to confirm these findings and refine predictive modelling for sternal wound reconstruction.

## Figures and Tables

**Table 1:** Demographics and Comorbidities.

Parameter	Total	Male	Female
Age, years (mean ± SD)	64.9 ± 11.9	64.0 ± 12.2	66.8 ± 11.4
Body weight, kg (mean ± SD)	89.0 ± 19.8	94.5 ± 18.1	77.2 ± 18.3
Height, cm (mean ± SD)	171.3 ± 16.1	176.4 ± 6.6	160.0 ± 23.6
BMI (body mass index), kg/m^2^ (mean ± SD)	29.9 ± 6.2	30.3 ± 5.4	29.2 ± 7.6
BSA(body surface area), m^2^ (mean ± SD)	2.0 ± 0.3	2.1 ± 0.2	1.8 ± 0.3
History of smoking	86 (51.8%)	64 (56.1%)	22 (42.3%)
Active smokers	36 (21.7%)	27 (23.7%)	9 (17.3%)
Arterial hypertension	153 (92.2%)	105 (92.1%)	48 (92.3%)
Dyslipidemia	129 (77.7%)	88 (77.2%)	41 (78.8%)
Diabetes mellitus	78 (47.0%)	48 (42.1%)	30 (57.7%)
Coronary heart disease (CHD)	115 (69.3%)	84 (73.7%)	31 (59.6%)
Previous myocardial infarction (MI)	75 (45.2%)	54 (47.4%)	21 (40.4%)
Percutaneous coronary intervention (PCI)	57 (34.3%)	45 (39.5%)	12 (23.1%)
Atrial fibrillation/flutter	38 (22.9%)	26 (22.8%)	12 (23.1%)
Peripheral arterial disease	40 (24.1%)	30 (26.3%)	10 (19.2%)
ICD (implantable Cardioverter Defibrillator) device	5 (3.0%)	5 (4.4%)	0 (0.0%)
Pacemaker	9 (5.4%)	7 (6.1%)	2 (3.8%)
Pulmonary disease	62 (37.3%)	47 (41.2%)	15 (28.8%)
Chronic lung disease	60 (36.1%)	45 (39.5%)	15 (28.8%)
Renal insufficiency	45 (27.1%)	30 (26.3%)	15 (28.8%)
Chronic dialysis	21 (12.7%)	13 (11.4%)	8 (15.4%)

Values are expressed as mean ± SD or n (%). Percentages refer to the respective sex group (n = 166).

**Table 2:** Preoperative Laboratory Parameters.

Parameter	Total	Male	Female
Hemoglobin (g/dL)	12.7 ± 2.5	13.0 ± 2.7	11.9 ± 1.8
Hematocrit (%)	38.2 ± 5.8	39.4 ± 5.7	35.6 ± 5.0
Platelets (×10^3^/µL)	225.3 ± 79.7	218.4 ± 75.2	240.4 ± 87.7
Leukocytes (×10^3^/µL)	8.9 ± 3.4	9.4 ± 3.6	7.7 ± 2.3
CRP (mg/dL)	1.8 ± 3.5	1.7 ± 3.4	2.1 ± 3.6
Albumin (g/L)	39.6 ± 5.6	40.2 ± 5.2	38.5 ± 6.4
Creatinine (mg/dL)	1.5 ± 1.4	1.5 ± 1.4	1.4 ± 1.4
LDH (U/L)	257.5 ± 213.8	250.6 ± 205.5	272.6 ± 232.1
eGFR (mL/min/1.73 m^2^)	83.0 ± 41.8	86.5 ± 42.3	75.1 ± 39.8
EuroSCORE (additive)	7.4 ± 3.7	7.0 ± 3.5	8.3 ± 4.1
EuroSCORE (logistic) %	13.7 ± 15.2	12.1 ± 12.8	17.2 ± 19.1
EuroSCORE II (%)	7.5 ± 10.6	6.1 ± 8.3	10.6 ± 14.1
STS mortality (%)	11.2 ± 104.2	2.4 ± 2.6	29.3 ± 181.6
STS morbidity and mortality (%)	133.4 ± 1008.6	14.0 ± 10.3	377.2 ± 1746.3

Values are expressed as mean ± SD. Percentages refer to the respective sex group (n = 166).

**Table 3:** Surgical Procedures and Postoperative Course.

Parameter	Total	Male	Female
Coronary artery bypass grafting (CABG)	108 (65.1%)	79 (69.3%)	29 (55.8%)
Valve procedures	33 (19.9%)	17 (14.9%)	16 (30.8%)
Aortic surgery	58 (34.9%)	37 (32.5%)	21 (40.4%)
Concomitant procedures	42 (25.3%)	28 (24.6%)	14 (26.9%)
Atrial fibrillation surgery	12 (7.2%)	9 (7.9%)	3 (5.8%)
Previous cardiac surgery	29 (17.5%)	24 (21.1%)	5 (9.6%)
ECMO required (≤30 days postop)	18 (10.8%)	13 (11.4%)	5 (9.6%)
ICU stay (days)	22.3 ± 37.7 (6.0; 0–253)	24.0 ± 40.1 (7.0; 0–253)	18.7 ± 31.9 (5.0; 1–161)
Total hospital stay (days)	67.5 ± 55.4 (50.5; 8–259)	63.7 ± 53.7 (48.0; 8–259)	75.7 ± 58.8 (56.0; 12–252)
Cardiopulmonary bypass (CPB) time (min)	147.7 ± 68.6 (133.5; 0–504)	152.1 ± 65.5 (138.0; 22–504)	138.3 ± 74.7 (122.0; 0–411)
Aortic cross-clamp (ACC) time (min)	83.9 ± 45.2 (76.0; 0–305)	84.8 ± 42.0 (79.0; 0–240)	81.7 ± 51.9 (71.0; 0–305)

Values are expressed as n (%) or mean ± SD (median; range). Percentages refer to the respective sex group (n = 166).

**Table 4:** Comparison Between Definitive Rewiring and PMF Reconstruction.

Parameter	Rewiring (n = 100)	PMF (n = 56)
Age, years (mean ± SD)	63.2 ± 12.1	67.4 ± 11.2
BMI, kg/m^2^ (mean ± SD)	29.4 ± 5.8	31.1 ± 6.4
EuroSCORE (additive)	8.0 ± 3.5	8.8 ± 3.9
EuroSCORE (logistic) %	11.3 ± 8.4	12.7 ± 8.8
EuroSCORE II (%)	5.4 ± 4.9	5.8 ± 5.7
History of smoking	49 (49.0%)	32 (57.1%)
Active smokers	18 (18.0%)	9 (16.1%)
Diabetes mellitus	12 (12.0%)	19 (33.9%)
Renal insufficiency	9 (9.0%)	14 (25.0%)

## Data Availability

The data underlying this article will be made available by the corresponding author upon request.
